# Better tolerance to Huanglongbing is conferred by tetraploid Swingle citrumelo rootstock and is influenced by the ploidy of the scion

**DOI:** 10.3389/fpls.2022.1030862

**Published:** 2022-11-03

**Authors:** Gary Sivager, Leny Calvez, Saturnin Bruyere, Rosiane Boisne-Noc, Barbara Hufnagel, Gerardo Cebrian-Torrejon, Antonio Doménech-Carbó, Olivier Gros, Patrick Ollitrault, Raphaël Morillon

**Affiliations:** ^1^ Centre de coopération Internationale en Recherche Agronomique pour le Développement (CIRAD), Unité Mixte de Recherche Amélioration Génétique et Adaptation des Plantes méditerranéennes et tropicales (UMR AGAP) Institut, Equipe Structure Evolutive des Agrumes, Polyploïdie et Amélioration Génétique (SEAPAG), F-97170 Petit-Bourg, Guadeloupe, French West Indies—Unité Mixte de Recherche Amélioration Génétique et Adaptation des Plantes méditerranéennes et tropicales (UMR AGAP) Institut, Univ. Montpellier, Centre de coopération Internationale en Recherche Agronomique pour le Développement (CIRAD), Institut National de Recherche pour l'Agriculture, l'Alimentation et l'Environnement (INRAE), Institut Agro, Montpellier, France; ^2^ Connaissance et Valorisation: Chimie des Matériaux, Environnement, Energie (COVACHIM-M2E) Laboratory Equipe Associée (EA) 3592, Unité de Formations et de Recherche (UFR) des Sciences Exactes et Naturelles, Université des Antilles, Pointe-à-Pitre, Guadeloupe; ^3^ Departament de Química Ananlítica, Facultat de Química, Universitat de València, Valencia, Spain; ^4^ Centre commun de caractérisation des matériaux des Antilles et de la Guyane (C3MAG), Unité de Formations et de Recherche (UFR) des Sciences Exactes et Naturelles, Université des Antilles, Pointe-à-Pitre, Guadeloupe; ^5^ Institut de Systématique, Evolution, Biodiversité, Muséum National d’Histoire Naturelle, Centre National de la Recherche Scientifique (CNRS), Sorbonne Université, École Pratique des Hautes Etudes (EPHE), Université des Antilles, Campus de Fouillole, Pointe-à-Pitre, France

**Keywords:** citrus, detoxification, Huanglongbing (HBL), lime, polyploidy, rootstock

## Abstract

Huanglongbing (HLB) is a disease that is responsible for the death of millions of trees worldwide. The bacterial causal agent belongs to *Candidatus* Liberibacter spp., which is transmitted by psyllids. The bacterium lead most of the time to a reaction of the tree associated with callose synthesis at the phloem sieve plate. Thus, the obstruction of pores providing connections between adjacent sieve elements will limit the symplastic transport of the sugars and starches synthesized through photosynthesis. In the present article, we investigated the impact of the use of tetraploid Swingle citrumelo (*Citrus paradisi* Macfrad × *Poncirus trifoliata* [L.] Raf) rootstock on HLB tolerance, compared to its respective diploid. HLB-infected diploid and tetraploid rootstocks were investigated when grafted with Mexican and Persian limes. Secondary roots were anatomically studied using scanning electron microscopy (SEM) and transmission electron microscopy (TEM) to observe callose deposition at the phloem sieve plate and to evaluate the impact of the bacterium’s presence at the cellular level. Voltammetry of immobilized microparticles (VIMP) in roots was applied to determine the oxidative stress status of root samples. In the field, Mexican and Persian lime leaves of trees grafted onto tetraploid rootstock presented less symptoms of HLB. Anatomical analysis showed much stronger secondary root degradation in diploid rootstock, compared to tetraploid rootstock. Analysis of the root sieve plate in control root samples showed that pores were approximately 1.8-fold larger in tetraploid Swingle citrumelo than in its respective diploid. SEM analyses of root samples did not reveal any callose deposition into pores of diploid and tetraploid genotypes. VIMP showed limited oxidative stress in tetraploid samples, compared to diploid ones. These results were even strongly enhanced when rootstocks were grafted with Persian limes, compared to Mexican limes, which was corroborated by stronger polyphenol contents. TEM analysis showed that the bacteria was present in both ploidy root samples with no major impacts detected on cell walls or cell structures. These results reveal that tetraploid Swingle citrumelo rootstock confers better tolerance to HLB than diploid. Additionally, an even stronger tolerance is achieved when the triploid Persian lime scion is associated.

## Introduction

Huanglongbing (HLB) is one of the most severe citrus diseases in the world and is caused by a phloem-restricted alphaproteobacterium (*Candidatus* Liberibacter spp.) (Bové, 2006). Three species of this gram-negative bacteria have been described in the taxonomy *Candidatus* Liberibacter asiaticus (CLas) (Bové, 2006), *Ca.* L. africanus (Jagoueix et al., 1994), and *Ca.* L. americanus (Teixeira et al., 2005). *Diaphorina citri* and *Trioza erytreae*, vectors of these species, spread the disease effectively. Economic losses are important (Santivañez et al., 2013; Neupane and Moss, 2016) because HLB leads to a production decrease due to impairments in tree development and fruit quality (Dala-Paula et al., 2019; Koh et al., 2020) and, eventually, tree- death. One of the most striking symptoms is observed on the leaves, which show asymmetrical yellow mottling on both sides of the midrib (Bové, 2006). Many of the symptoms of HLB are due to physiological disturbances related to the strong biosynthesis of callose at the phloem sieve plate, thus preventing the flow of elaborated sap (Achor et al., 2010; Koh et al., 2012). As a result, photosynthetic products, such as starch, accumulate in the leaves (Etxeberria et al., 2009; Achor et al., 2010). The accumulations of sugars, amino acids, fatty acids, and secondary metabolites were shown to be significantly remodeled depending on the tissues and species investigated (Chin et al., 2014; Padhi et al., 2019; Chen et al., 2022). These foliar symptoms, although they are the most visible, are rather late symptoms because they develop 6 months or more after those at the root level (Graham et al., 2013). Although many studies focused on the implication of HLB in the canopy, research on the root system is still limited. Johnson et al. (2013) showed that the root system was a bacterial reservoir. Indeed, the bacterial quantity seemed more important at the root level than it was at the foliar level. Moreover, a relation between the decrease of the bacterial quantity in roots and the appearance of new flushes indicated that the roots were a place of multiplication and development for the bacteria to colonize the rest of the plant. Different studies (Johnson et al., 2013; Achor et al., 2020) demonstrated that, unlike the canopy, no callose deposition could be observed at the phloem sieve plate to explain why the roots would be the bacterial reservoir. Finally, HLB-infected plants show a strong oxidative stress, inducing detoxification mechanisms in order to eliminate the reactive oxygen species (ROS) produced in excess (Martinelli et al., 2016). ROS, which are free radicals and non-radical molecules, are key components of the network of signaling pathways and act as major regulators of plant cell physiology and cellular responses to environmental factors (Bhattacharjee, 2012). Likewise, families of molecular compounds, such as polyphenols, are useful in the defense against environmental stress. It has been shown that these compounds also have antioxidant effects (Agati et al., 2012; Kumar and Pandey, 2013) in order to reduce the cell toxicity of ROS. Therefore, following a stress, the concentration of phenolic compounds increases to fight against it (Sharma et al., 2019).

It has long been considered that there is no genetic resistance to HLB in genus *Citrus*. However, important variabilities of behavior under HLB constraints have been reported for the different species of the genus *Citrus* (Stover et al., 2015; Miles et al., 2017), which should be associated with better adaptation to the bacteria or differential attractiveness to the vector. For the related genera *Poncirus*, the lower susceptibility of trifoliate orange could be related to a lower leaf appetence of *Diaphorina citri*, which, in turn, would limit the possibilities of infection and development of CLas (Westbrook et al., 2011; Richardson and Hall, 2013). More recently, the evaluation of HLB symptoms in germplasm collections under HLB constraints revealed complete resistance to HLB in related genera and, particularly, in Australian citrus species (Ramadugu et al., 2016; Alves et al., 2021). Several HLB-tolerant citrus hybrids and relatives were shown to synthetize antimicrobial peptides that can inhibit infections by CLas (Blaustein et al., 2018; Huang et al., 2021).

Furthermore, studies have shown that polyploid plants are generally more tolerant to various biotic and abiotic stresses (for review, see Ruiz et al., 2020). For example, the effectiveness of citrus tetraploid (4x) rootstocks has been shown against water deficit (Allario et al., 2013). In a recent work, it was reported that triploid varieties, such as Persian lime, have better anatomical and physiological properties than diploid (2x) species do. These present a better antioxidant mechanism, explaining why triploids behave much better in the field regarding HLB disease (Sivager et al., 2021). Other studies suggested that some 4x rootstocks could limit the impact of HLB (Grosser and Gmitter, 2011; Grosser et al., 2012). However, there are no published studies specifically analyzing the impact of rootstock polyploidy on HLB.

In this study, a comparison of the behavior of the 2x Swingle citrumelo Swingle 4475 rootstock with its respective 4x form was performed. The 2x Swingle citrumelo Swingle 4475 resulted from a cross between a grapefruit Duncan and *Poncirus trifoliate*, and the 4x was selected in seedlings of 4475 Swingle citrumelo because of chromosome duplication in nucellar tissue (Aleza et al., 2011). Thus, it was possible to limit the impact of other environmental factors. Additionally, in order to visualize the different anatomical features in field conditions and possible callose deposits, a collect of symptomatic samples on trees naturally infected by psyllids in the field was done. Different analysis by microscopy were performed to quantify the bacteria in the 2x and 4x rootstocks. Finally, quantification of polyphenols associated with an electrochemical activity to determine the antioxidant power of these two genotypes under HLB stress was done. Altogether, these findings provide insights regarding the determinants associated with better HLB tolerance in 4x Swingle citrumelo rootstock. Furthermore, these results underline the strong interaction between rootstock and scion, influencing the tolerance to this disease.

## Materials and methods

### Plant material and growth condition

The INRAE-CIRAD of San Giuliano in Corsica (France) provided 2x and 4x Swingle citrumelo 4475 (SRA 928; *Citrus paradisi* Macfrad × *Poncirus trifoliata* [L.] Raf) seeds from the collection of the “CRB Citrus” biological resource center (Luro et al., 2017). Seedlings preparation and analysis were performed according to Sivager et al. (2021).

A total of sixty genetically conform and uniform seedlings (2x and 4x) were selected for further investigation. Twenty 10-month-old 2x and 4x rootstock seedlings were grafted using budwoods collected on asymptomatic HLB leaves and control budwoods of the 2x Mexican lime (*Citrus aurantiifolia* [Christm. Swingle, SRA 140]) and the 3x Persian lime (*Citrus latifolia* [Yu. Tanaka] Tanaka, SRA 58). Among them, six control and infected trees of each genotype were grown according to Sivager et al. (2021). Twenty-eight to 38 months old trees were used for physiological, microscopic, and biochemical investigations. Field observations were also performed on 4-year-old trees of the same rootstock and variety combinations naturally infected for 2 years.

### HLB monitoring and roots preparation for ultrastructural analysis (SEM and TEM)

HLB monitoring and roots preparation for scanning electron microscopy (SEM) were performed according to Sivager et al. (2021).

Transmission electron microscopy (TEM). Fresh longitudinal sections of roots were prefixed overnight at 4°C in 2.5% glutaraldehyde in a phosphate saline buffer (PBS; pH 7.2), then for 1 hour under vacuum at RT. Samples were rinsed twice in the same buffer for 10 minutes in order to remove aldehyde before fixation of 45 minutes at room temperature in 1% osmium tetroxide in PBS. Then, samples were rinsed three times in distilled water for 5 minutes and post-fixed with 2% aqueous uranyl acetate for one more hour. After three rinses in distilled water, specimens were dehydrated through a graded acetone series at room temperature before embedding in LR white resin under vacuum. Thin sections (80 nm thick) were observed in a Tecnai G20 TEM at 200 Kv or by using STEM mode with a FEI Quanta 250 electron microscope at 20 kV.

### Root preparation for blue aniline staining, fluorescence *in situ* hybridization (FISH) of Candidatus Liberibacter asiaticus in the roots of 2x and 4x Swingle citrumelo rootstocks grafted with Mexican or Persian limes

Aniline staining and fluorescence *in situ* hybridization (FISH) were performed according to Sivager et al. (2021).

### Polyphenols contents

Roots were fixed with liquid nitrogen and then ground. Then, 500 mg of ground samples were incubated for 72 h in 10 mL 100% methanol, before filtering. The polyphenolic assays were performed with “Folin Ciocalteau Phenolic Content Quantification Assay” kit according to the manufacturer’s instructions (Bioquochem). A standard curve was done to determine the sample phenolic concentrations.

### Electrochemistry

Electrochemistry was done according to the method of Palmeira-Mello et al. (2021), with a Bio-Logic SP-300 tool. Electrochemical experiments were performed at 298 ± 1 K in a conventional three-electrode cell, using a platinum wire auxiliary electrode and an Ag/AgCl (3M NaCl) reference electrode. Measurements were carried out with a Bio-Logic SP-300 equipment, using 0.10 M potassium phosphate buffer at pH 7.0 as a supporting electrolyte. The working electrode was prepared by evaporating 50 μL of a methanol of ground root extract samples under air on a glassy carbon electrode (GCE, BAS MF 4012, geometrical area 0.071 cm^2^). To mimic the natural environment, no electrolyte degasification was performed. Three biological repetitions were done for each sample. Measurements were performed from -1.5 V to 1.5 V (red/ox), 1.5 V to -1.5 V (ox/red), and 0 to 1.5 V (short ox).

### Statistical analysis

For each given date of the experiment, data were subjected to variance analyses using a one-way analysis of variance (ANOVA; SigmaPlot version 11, from Systat Software, Inc., San Jose California USA; www.systatsoftware.com). One-way ANOVA, followed by Tukey’s *post hoc* test, was used to assess significant differences. Statistical significance was set at *P ≤* 0.05.

## Results

### Impact of the disease in field condition on the different scion/rootstock associations

In the field, when grafted onto 2x Swingle citrumelo, 4-year-old Persian lime trees show about 30% higher growth, compared to Mexican lime trees. When grafted onto 4x Swingle citrumelo rootstock, Persian lime and Mexican lime trees showed more limited growth, compared to tree associations with 2x Swingle citrumelo: 4 years after planting and 2 years after natural HLB infection, tree height onto 4x rootstock was approximately 20% lower. Leaf mottling induced by HLB was quite similar in Persian lime leaves whatever the rootstock ploidy. However, leaf yellowing in Mexican lime trees was more limited when grafted onto 4x Swingle citrumelo, compared to 2x Swingle citrumelo. SPAD and effective quantum yield of PSII values measured in symptomatic leaves of Mexican and Persian leaves grafted onto 4x Swingle citrumelo, compared to the respective 2x Swingle citrumelo, were higher (**Table 1**). Also fruit production in the third year was estimated and showed a trend with a higher production when grown onto scions are grated onto 4x Swingle citrumelo. Also fruit weight was significantly increased for both genotypes when grafted onto 4x Swingle citrumelo (**Table 1**)

**Table 1:** Leaf physiological characterization of Mexican and Persian limes. Leaf greenness (SPAD unit), effective quantum yield of PSII (ΦPSII) within light-adapted leaves, measured in symptomatic leaf from Mexican and Persian limes, grafted onto 2x and 4x Swingle citrumelo rootstocks, respectively.

Scion	Mexican lime		Persian lime	
Rootstock	Swingle citrumelo 2x	Swingle citrumelo 4x	Swingle citrumelo2x	Swingle citrumelo 4x
**SPAD (AU)**	41.5 ± 5.98a	52.42 ± 4.6ab	56.28 ± 11.94b	59.13 ± 9.43b
**QY (AU)**	0.36 ± 0.13a	0.47 ± 0.01b	0.50 ± 0.13b	0.59± 0.06c
**Fruit production (kg)**	20.78 ± 2.35a	32.84 ± 4.11b	24.26 ± 4.71ab	27.38 ± 5.1ab
**Fruit weight** **(g)**	35.1 ± 0.8a	45.1 ± 1.75b	83.7 ± 1.65c	108.7 ± 2.6d

Fruit production and fruit weight after three years of plantation for the different scion/rootstock associations are also presented. Results are expressed as mean +SE (n = 5 to 20 measurements). ANOVA tests were performed to determine if HLB led to significant differences. Data with a different letter are statistically different. Results with Swingle citrumelo 2x are extracted from Sivager et al. (2021).

#### Anatomical impact of Huanglongbing in 2x and 4x rootstock

Investigations of phloem cells of CLas+ Mexican and Persian lime grafted onto 4x Swingle citrumelo were performed. In asymptomatic petiole leaves, the presence of callose and the plugging of the pores were similar to the Mexican lime and Persian grafted onto 2x Swingle citrumelo (**Suppl. Figure 1**). The infections by HLB in control and infected trees grown in greenhouse conditions were tested by qPCR. As expected, controls were negative, and trees infected by HLB were positive (**Table 2**). For infected trees, the titer of the bacterium of the bacterium using the Ct value quantified in 2x and 4x Swingle citrumelo rootstocks remained non-determined, as if the bacterium was not present independently of the ploidy level of the scion grafted on it. Investigations of the bacterial load at root level remain limited, and we did not find literature on CLas qPCR in Swingle citrumelo roots to compare with our results. Thus, a quantification of CLas in sour orange root has been done, which was previously shown to have high titer (Park et al., 2018). In that case, CLas titer was high in the petiole, with Cts being close to 18. The unexpected results obtained in 2x and 4x Swingle citrumelo may be explained by an absence of the bacterium or an inhibition of the qPCR. This last hypothesis is supported by FISH analysis using a probe targeted against CLas on the same amount of ground root material. Interestingly, fluorescence in the root of 2x Swingle citrumelo grafted with Persian lime was much higher than in root of 2x Swingle citrumelo grafted with Mexican lime (**Suppl. Figures 2C, E**). When grafted onto 4x Swingle citrumelo, the observed fluorescence was more limited (**Suppl. Figures 2D, F**). For CLas+ asymptomatic (AS) petiole leaves, the titer of the bacterium quantified in Persian lime trees was much lower than in Mexican lime trees (Ct of 28 versus 23; **Table 2**). Nevertheless, the use of 4x rootstock did not affect the titer of the bacterium in petioles.

**Table 2:** Quantitative real-time PCR in roots of 2x and 4x Swingle citrumelo and petioles from Mexican and Persian limes of the control (CLas-) and infected samples (CLas+).

Rootstock	Scion	CLas-		CLas+	
		Root	Petiole	Root	Petiole
Swingle citrumelo 2x	Mexican lime	ND	ND	ND	22.58 ± 0.73a
Swingle citrumelo 2x	Persian lime	ND	ND	ND	26.51 ± 0.69b
Swingle citrumelo 4x	Mexican lime	ND	ND	ND	22.85 ± 0.45a
Swingle citrumelo 4x	Persian lime	ND	ND	ND	27.81 ± 0.30b

For petioles, asymptomatic leaves were selected. Ct results are expressed as mean +SE (n = 3) and ND means Not Determined. Data with a different letter are statistically different.

**Figure 1:**
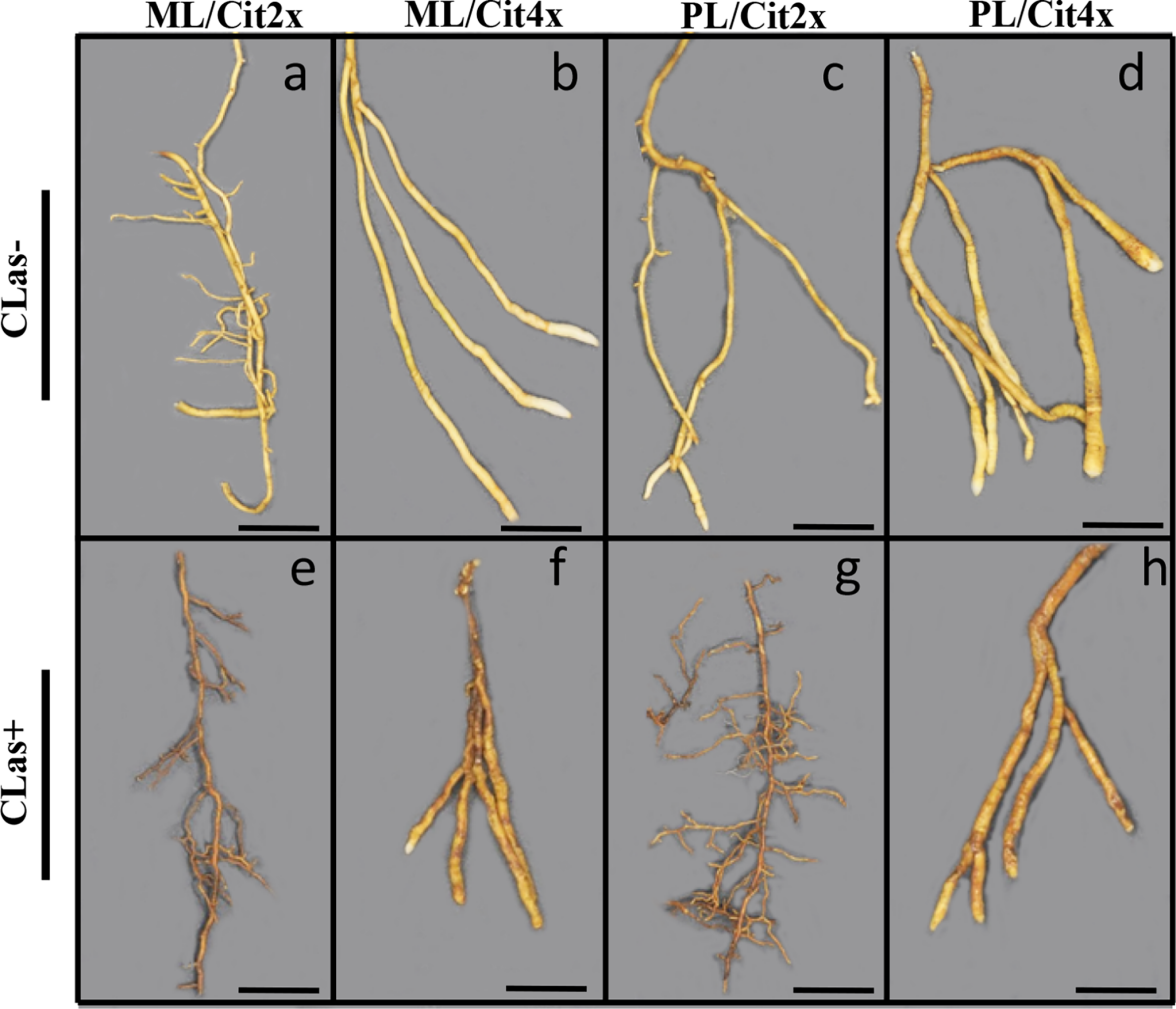
Macroscopic pictures of 2x **(A, C, E, G)** and 4x **(B, D, F, H)** Swingle citrumelo rootstocks secondary roots in control (CLas-) and HLB infected (CLas+) trees. ML and PL mean Mexican lime ad Persian lime. Cit2x and Cit4x mean 2x Swingle citrumelo and 4x Swingle citrumelo. Bars = 1 cm.

**Figure 2:**
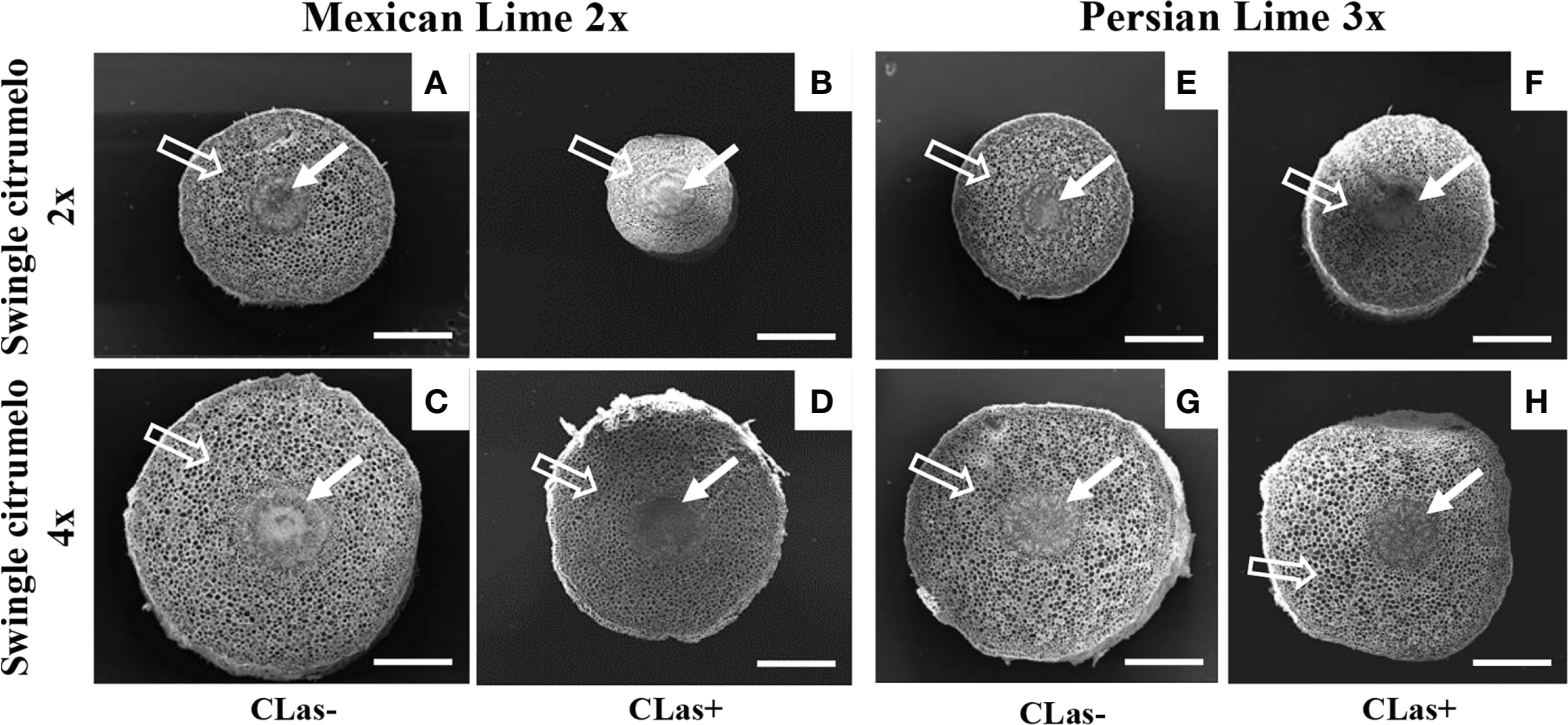
Transversal SEM pictures of 2x **(A, B, E, F)** and 4x **(C, D, G, H)** roots of Swingle citrumelo rootstocks grafted with 2x or 3x limes infected or not by CLas. Full white arrows show the central cylinder (phloem & xylem) of the root, and empty white arrows show the root cortex. Bars = 0.5 mm.

**Figure 3:**
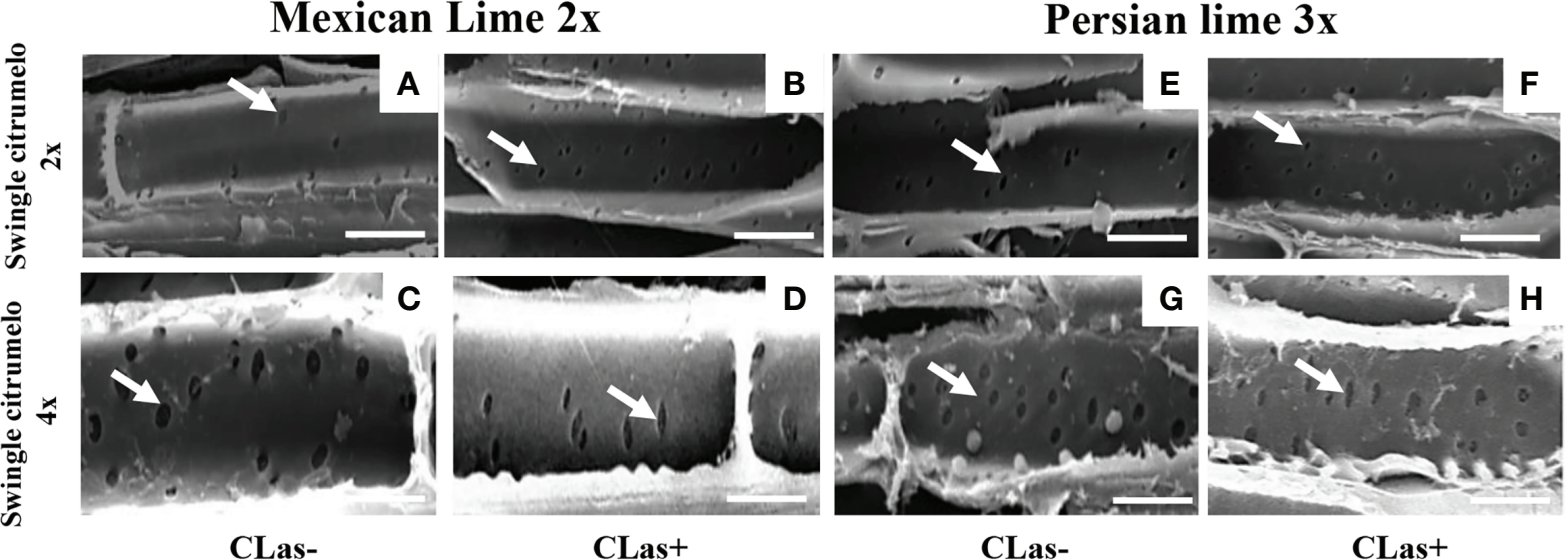
Longitudinal SEM of 2x **(A, B, E, F)** and 4x **(C, D, G, H)** root phloem cells of Swingle citrumelo rootstocks grafted with 2x or 3x limes infected (CLas+) or not (CLas-). White arrows indicate the pores in the phloem cell wall. Bars = 15 µm.

**Figure 4:**
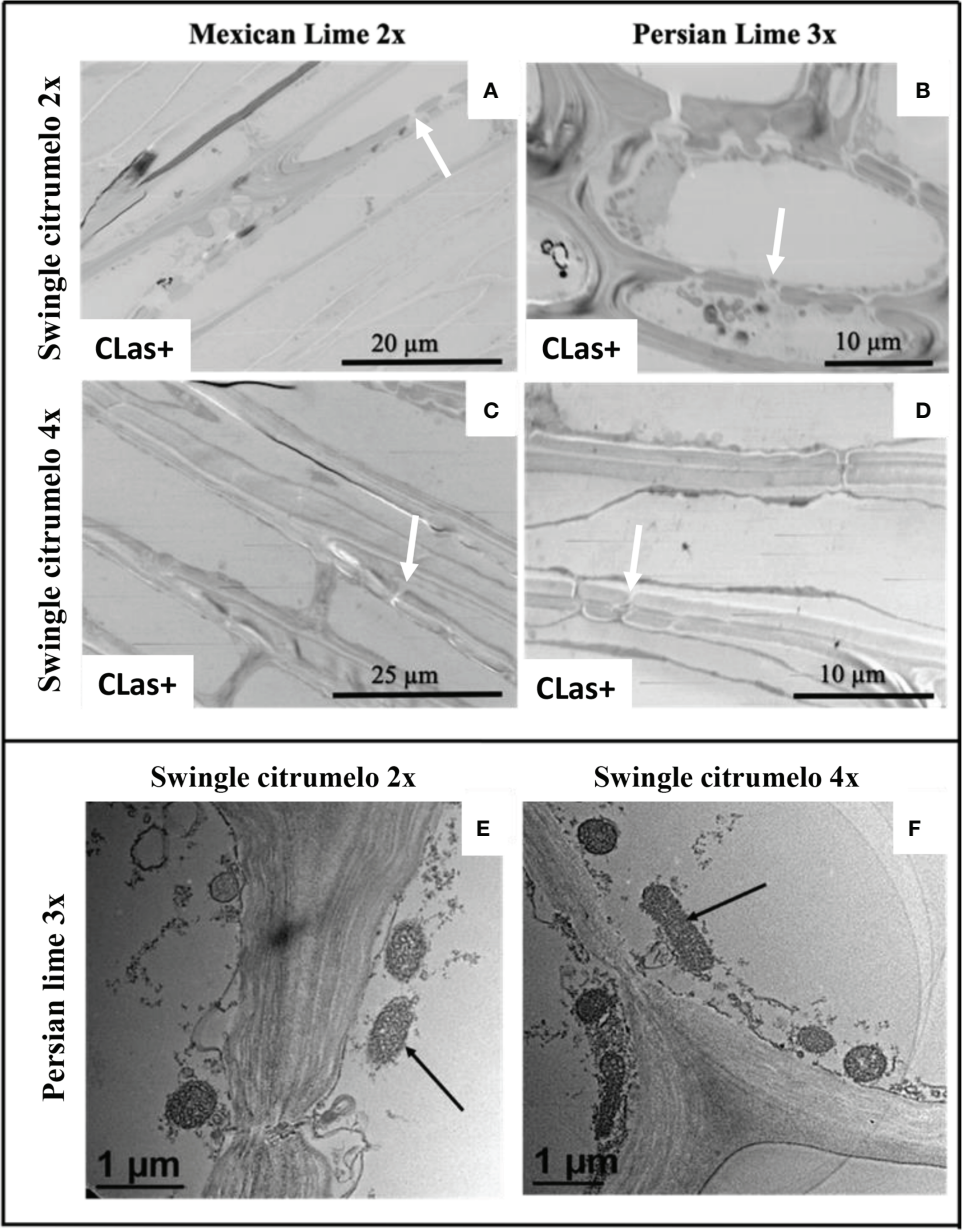
Transmission electron microscopy of phloem sieve plate in 2x **(A, B)** and 4x **(C, D)** roots of Swingle citrumelo grafted with Mexican lime and Persian lime infected (CLas+) by CLas. White arrows in pictures **(A-D)** indicate pores in the cell wall that are opened and not plugged by callose **(A–C)**. Visualization of CLas (black arrows) in 2x **(E)** and 4x **(F)** CLas+ root samples of Swingle citrumelo grafted with Persian lime.

Investigations were also performed at the microscopic and biochemical levels on trees grown in greenhouse conditions. Macroscopic pictures of secondary roots of CLas- and CLas+ rootstocks showed a browning of the root color due to the disease (**Figure 1**). Tetraploid Swingle citrumelo secondary roots are thicker than 2x Swingle citrumelo, with more limited numbers of very fine roots. Infection induced a degradation of fines roots in 2x Swingle citrumelo (**Figures 1E, G versus C, A**), whereas limited changes were observed in 4x Swingle citrumelo (**Figures 1F, H versus D, B**). To decipher the disease’s impact in the phloem, scanning microscopy analyses were performed in the leaf petioles of the different genotypes. The root diameters and areas were greater in 4x Swingle citrumelo, than these were in 2x Swingle citrumelo (**Table 3**, **Figure 2**). In CLas- and CLas+ samples, the cortex, the phloem area, the xylem area, and the central cylinder were also greater in 4x Swingle citrumelo, compared to 2x Swingle citrumelo. Interestingly, the infection caused a tissue and vessel collapse in the 2x secondary roots of the Mexican lime/2x Swingle citrumelo association tree but not in the 2x secondary roots of the Persian lime/2x Swingle citrumelo association (**Figures 2B versus F**; **Table 3**). Transversal SEM of 2x and 4x Swingle citrumelo secondary roots did not allow us to see the pores inside the walls of phloem cells (**Suppl. Figure 3**). Thus, longitudinal SEM were performed in CLas- and CLas+ secondary roots of 2x and 4x Swingle citrumelo (**Figure 3**). For the CLas- and CLas+ secondary roots, 2x Swingle citrumelo presented phloem cells and pores between cells that were smaller than in Swingle citrumelo 4x (**Table 3**; **Figure 3**). No callose deposition and starch grains was observed in CLas+ samples. Aniline blue staining in the same samples confirmed the absence of specific staining in the phloem and thus callose deposition in infected 2x and 4x secondary roots (**Suppl. Figure 4**). Investigations were performed by TEM in order to visualize the presence of CLas and its possible implication at the cellular level. Cross sections of phloem cells in CLas- samples indicated the presence of the pores allowing the transfer of the sap between cells (**Figures 4A–F**). As expected in 4x root samples, cell walls were thicker, with a mean thickness of 1104 ± 65 µm, compared to 654 ± 39 µm for 2x roots (**Figures 4A–D** respectively). In our hands, CLas was visible in CLas+ samples of 2x and 4x Swingle citrumelo rootstocks grafted with Mexican lime and Persian limes. **Figures 4E, F** illustrates the presence of CLas in 2x and 4x CLas+ Swingle citrumelo rootstocks grafted with Persian lime. CLas was present close to the pores and the cell walls. Degradation of cell walls was not obvious in 2x Swingle citrumelo rootstocks when compared to 4x.

**Table 3:** Anatomical characterization of 2x or 4x Swingle citrumelo Swingle rootstock grafted with Mexican lime or Persian lime, infected (CLas+) or not (CLas-) by *Candidatus* Liberibacter asiaticus.

	CLas-				CLas+				
	Mexican lime		Persian lime		Mexican lime		Persian lime		
	Cit 2x	Cit 4x	Cit 2x	Cit 4x	Cit 2x	Cit 4x	Cit 2x		Cit 4x
**Roots diameter** **(mm ± SE)**	2.0 ± 0.3 b	3.2 ± 0.2 c	2.1 ± 0.2 b	3.6 ± 0.2 c	1.3 ± 0.1 a	3.7 ± 0.2 c	1.9 ± 0.2 b	3.5 ± 0.1 c	
**Roots area (µm^2^ ± SE)**	1844228 ± 137256 b	4061130 ± 5525 c	2282794 ± 650487 b	4418763 ± 387606 c	1245116 ± 169565 a	4456783 ± 512842 c	1730391 ± 187135 ab	43188613 ± 430822 c	
**Cortex area (µm^2^ ± SE)**	1638197 ± 117490 b	3493397 ± 155576 d	2000482 ± 572185 bc	3847399 ± 246901 d	1007908 ± 147775 a	4460485 ± 314249 d	1912855 ± 49466 c	3712759 ± 350772 d	
**Phloem area (µm^2^ ± SE)**	50117 ± 7018 ab	168679 ± 48079 d	66560 ± 16565 b	100230 ± 6637 c	42938 ± 3384 a	240533 ± 33692 d	72616 ± 2909 b	163728 ± 20463 d	
**Phloem cell area (µm^2^ ± SE)**	444 ± 43 a	637 ± 59 b	458 ± 18 a	705 ± 62 b	890 ± 103 b	804 ± 64 b	839 ± 97 b	877 ± 106 b	
**Area of the pores of phloem cells (µm^2^ ± SE)**	1.1 ± 0.2 a	2.1 ± 0.1 b	1.4 ± 0.1 a	2.2 ± 0.2 b	1.5 ± 0.1 a	2.2 ± 0.1 b	1.2 ± 0.1 a	2.3 ± 0.1 b	
**Xylem area (µm^2^ ± SE)**	65404 ± 3574 a	260109 ± 61723 c	126557 ± 19918 b	294223 ± 60531 c	67196 ± 2474 a	393654 ± 58604 c	111796 ± 5548 b	316340 ± 57269 c	
**Central cylinder area (µm^2^ ± SE)**	164687 ± 17597 a	404791 ± 80462 b	167617 ± 22661 a	387409 ± 24934 b	132275 ± 6643 a	494866 ± 66518 b	218919 ± 12855 a	429525 ± 31188 b	

Results are express as the mean ± SE (n = 8 to 25). ANOVA tests were performed to determine if HLB led to significant differences. Data with a different letter are statistically different. Cit2x means 2x Swingle citrumelo and cit4x means 4x Swingle citrumelo.

#### Electrochemical analysis of 2x and 4x secondary roots of Swingle citrumelo rootstocks infected by Huanglongbing

**Figure 5:**
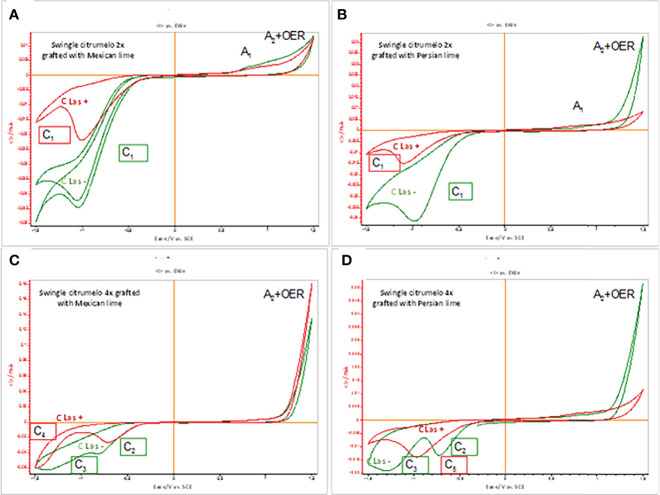
CV profiles of 2x or 4x roots of Swingle citrumelo rootstocks grafted with Mexican lime or Persian lime infected (CLas+, red curves) or not (CLas-, green curves). **(A)** Mexican lime/2x Swingle citrumelo; **(B)** Persian lime/2x Swingle citrumelo; **(C)** Mexican lime/4x Swingle citrumelo; **(D)** Persian lime/4x Swingle citrumelo. Potential scan initiated at 0.0 V in the negative direction; potential scan rate 10 mV s^−1^.

Cyclic voltammograms (CV) of roots of 2x and 4x Swingle citrumelo immersed in air-saturated aqueous phosphate buffer at pH 7.0 were studied. **Figure 5** compares the CV of glassy carbon electrode (GCE) in 2x (**Figures 5A, B**) or 4x (**Figures 5C, D**) Swingle citrumelo roots grafted with Mexican lime (**Figures 5A, C**) or Persian lime (**Figures 5B, D**) infected or not by HLB. For the analysis of grafted Swingle citrumelo reaction to the infection, we focused the study on the scanning of the potential in the negative direction (**Figure 5**). In the case of 2x Swingle citrumelo grafted with Mexican lime, healthy roots (**Figure 5A**, green curve) displayed a cathodic signal at ca. −1.0 V (C_1_) and a weak anodic wave (A_1_) ca. 0.7 V preceding a rising current at ca. 1.4 V corresponding to the second anodic signal (A_2_) superimposed to the oxygen evolution reaction (OER). The cathodic signal C_1_ is superimposed to the reduction of dissolved oxygen (C_ox_), as denoted by blank experiments at unmodified GCE. In the case of cathodic scan of infected rootstocks (**Figure 5B**, red curve), a similar reduction signal in the same region of potentials than C_1_ was detected with clearly lower intensity, while the anodic wave A_1_ becomes also lowered. Similar reduction signals were detected in the case of 2x Swingle citrumelo grafted with Persian lime (**Figure 5B**). However, this signal is dramatically depleted in the CLas+ rootstocks. **Figure 5C** presents the cyclic voltammograms recorded for GCEs modified with films of 4x Swingle citrumelo secondary roots grafted with Mexican lime. Upon scanning the potential in the negative direction, healthy roots present two cathodic signals at approximately −0.8 (C_2_) and −1.35 (C_3_) V. These signals appear at potentials clearly different from those for the signal C_1_ recorded with the 2x Swingle citrumelo CVs. The voltammograms of the infected rootstocks show a decrease in the intensity of the peak C_2_ and the disappearance of the peak C_3_, which is apparently substituted by a cathodic signal at −1.45 V(C_4_). Finally, cyclic voltammograms were recorded at GCEs modified with the films of roots of 4x Swingle citrumelo grafted with Persian lime (**Figure 5D**). Cathodic signals at approximately −0.8 (C_2_) and −1.35 (C_2_) V were recorded again. In CLas+ trees, a new signal at ca. −0.95 V (C_5_) appeared, replacing the signals of the healthy sample (C_2_ and C_3_). The signal C_4_ appearing at 4x Swingle citrumelo grafted with Mexican lime (**Figure 5C**) was entirely absent here; whereas, the strong A2 + OER signal was considerably decreased in 2x Swingle citrumelo grafted with Persian lime (**Figure 5B**). Interestingly, the C5 peak is essentially coincident with the peak C1 recorded for 2x forms (**Figures 1B**, **5A**).

#### Polyphenol content and tissue structure in 2x and 4x secondary roots of Swingle citrumelo rootstocks infected by Huanglongbing

**Figure 6:**
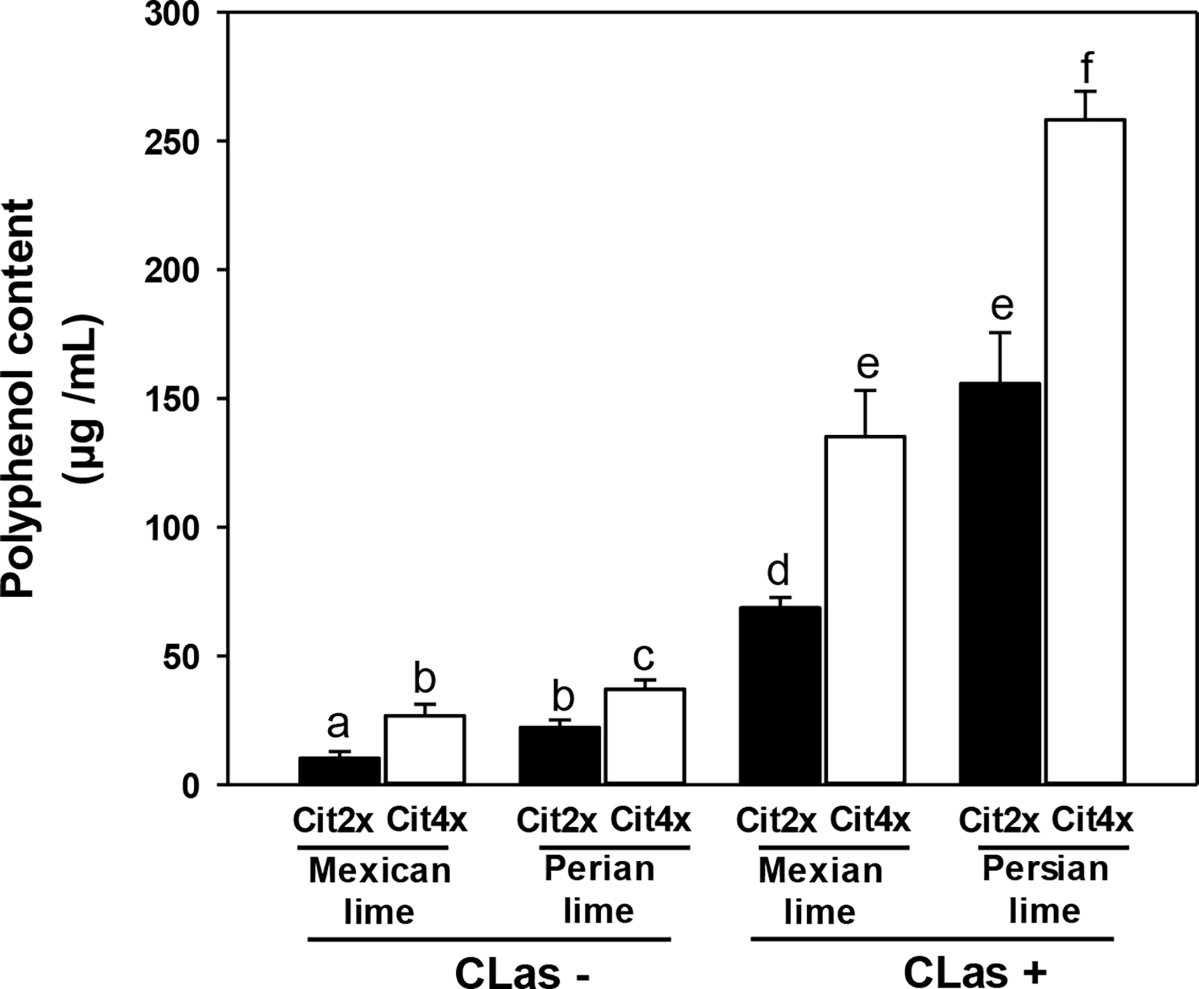
Polyphenols content in 2x and 4x roots of Swingle citrumelo rootstocks grafted with Mexican lime and Persian lime infected (CLas+) or not (CLas-). Cit2x and Cit4x mean 2x swingle citrumelo and 4x swingle citrumelo, respectively. Data with a different letter are statistically different. Vertical bars indicate the mean value ± SE.

Polyphenol contents were estimated in 2x and 4x Swingle citrumelo roots infected by HLB and in control samples. In CLas- the polyphenol contents were limited (<30 µg/mL) but were all higher in 4x rootstocks, compared to 2x rootstocks (**Figure 6**). Interestingly, trees grafted with Persian lime presented greater polyphenol contents in roots than when grafted with Mexican lime. In CLas+ root samples, polyphenols contents were dramatically increased. Tetraploid rootstocks displayed a greater increase, compared to 2x. Finally, the scion also influenced the polyphenols contents in CLas+ 2x and 4x Swingle citrumelo rootstocks: a two-fold increase of the polyphenol root contents were measured when grafted with Persian lime, compared to Mexican lime, regardless of the rootstock’s ploidy level (**Figure 6**).

## Discussion

### Anatomical differentiation between Mexican lime and Persian lime trees grafted on 2x and 4x Swingle citrumelo

In the field, when grafted onto Swingle citrumelo 2x, infected Persian lime scion showed higher growth, compared to infected Mexican lime scion (Sivager et al., 2021). Such phenotypic differentiation associated with polyploidy has been reported in numerous studies (for review, see Ruiz et al., 2020). Among them, one may indicate greater cell sizes, organ sizes, and higher leaf greenness (Mouhaya et al., 2010; Allario et al., 2011; Sivager et al., 2021). In our hands, when grafted onto 4x Swingle citrumelo compared to 2x Swingle citrumelo, the fruit production in the third year of plantation were similar or higher for Persian lime and Mexican lime. This result may be related to the beneficial impact of the ploidy of the rootstock regarding HLB since it had been previously reported that the tetraploidy of the rootstock would drastically limit the cumulative fruit production of clementine compared to the production onto the respective diploid (Hussain et al., 2012). Fruit weight was significantly higher for both limes when grafted onto 4x Swingle citrumelo compared to 2x Swingle citrumelo. Similar results were reported in citrus by Hussain et al. (2012) and in other plant species (Ruiz et al., 2020). When grafted onto 4x Swingle citrumelo, the height of Mexican lime and Persian lime scions were smaller than when grafted onto the respective 2x Swingle citrumelo, as previously observed (Hussain et al., 2012). Previous investigations showed that the polyploidy in rootstocks and canopies has an antagonist effect on photosynthetic and respiratory function. The photosynthetic activities of 4x seedlings (Allario et al., 2011) and 2x sweet orange grafted on 4x rootstocks (Allario et al., 2013) were shown to be reduced, compared to plants with 2x root systems. This reduction was associated with higher constitutive ABA biosynthesis in 4x roots (Allario et al., 2013). In the field, it has been also showed that the tree size reduction in clementine scion, which was related to the use of 4x rootstocks, was indeed related to more limited photosynthesis activity (Hussain et al., 2012).

The investigation of secondary roots of 2x and 4x Swingle citrumelo showed the 4x were thicker and less numerous than those in 2x (**Figures 1**, **2**, **Table 3**), as previously mentioned (Allario et al., 2011; Oliveira et al., 2017). Such a phenotype has been associated in 4x Swingle citrumelo with better tolerance to abiotic stress, such as water deficit, when investigated in potted conditions (Allario et al., 2013).

### Anatomical characterization at scion and secondary roots levels in 2x and 4x Swingle citrumelo grafted with Mexican and Persian lime cultivars grown under HLB stress

Previous observations in the field showed that the Persian lime trees were much less affected than Mexican lime trees regarding HLB, which fits other observations (Deng et al., 2019; Sivager et al., 2021). This tolerance was associated with the maintenance of vigorous growth and fruit yield. In greenhouse conditions, it has been observed that, when grafted onto 2x Swingle citrumelo, infected Mexican lime leaves were much more yellow than Persian lime leaves (Sivager et al., 2021). Interestingly, Mexican lime leaf yellowing and leaf mottling were more limited when grafted onto 4x Swingle citrumelo rootstock, suggesting a benefic impact regarding rootstock HLB on the scion. The greater values of SPAD and effective quantum yield of PSII measured in symptomatic leaves of Mexican and Persian limes grafted onto 4x Swingle citrumelo, compared to the respective 2x Swingle citrumelo, are in agreement with this hypothesis (**Table 1**). Koh et al. (2012) showed that HLB leads to increased callose synthesis in the phloem vessels, resulting in clogging of the pores at the sieve plate between cells (Achor et al., 2010; Sivager et al., 2021). The specific phenology of the Persian lime, with a slower plugging of phloem cell pores and a better phloem regeneration observed in Bearss lemon (Deng et al., 2019), is in agreement with a longer leaf lifetime of that genotype, compared to the Mexican lime genotype (Sivager et al., 2021). However, it is important to note that the genetic origin of the Persian lime (it is a hybrid between a Mexican lime and a lemon) may also explain in part the observed the better tolerance of that genotype as previously mentioned by Sivager et al. (2021). Investigation by SEM of CLas+ petiole asymptomatic phloem cells of Mexican and Persian limes grafted onto 4x Swingle citrumelo did not indicate a more limited clogging of the pores, compared to the same scions grafted onto 2x Swingle citrumelo, suggesting that the callose deposition was not impacted by the rootstock ploidy status. Thus, we investigated the impact of HLB at the root level. Interestingly, HLB induced a browning of the root color, with a degradation of fines roots in 2x Swingle citrumelo that was much more significant than it was in 4x Swingle citrumelo (**Figure 1**). To verify whether the 4x in the root may favor a better adaption to HLB due to more limited intercellular sieve pore plugging, we performed SEM. Transversal cross sections did not indicate the pores in the secondary root phloem cells because of their longer size and the limited chance to cut at the right place when preparing the samples (**Suppl. Figure 3**). Indeed, the length of phloem cells were often > 150 µm, favoring long distance transport of the sap without cell walls to cross. Longitudinal SEM of secondary roots confirmed that phloem cells and sieve plate pores of 4x Swingle citrumelo were larger than those of 2x Swingle citrumelo were. In HLB-infected secondary roots, the phloem cell sieve pores were not obstructed by callose deposition, whatever the ploidy status of the rootstock, as previously reported (Achor et al., 2020). According to these authors (Achor et al., 2020), the maintaining phloem sap flux due to non-callose deposition at the sieve plate of the phloem cells explains why the roots would be a bacterial reservoir. Thus, root degradation would more likely relate to a direct impact of the bacterium than to perturbation of sieve flux. Furthermore, better phloem regeneration in tolerant genotypes, as proposed by Deng et al. (2019), may also occur in 4x roots and may favor adaptation to HLB.

### Differential response to oxidative stress in Mexican and Persian limes

Oxidative stress genes coding for antioxidant enzymes were shown to be upregulated in HLB-susceptible citrus, compared to tolerant citrus (Tang and Vashisth, 2020), which induces mature-fruit drop in HLB-infected citrus trees. Previous results showed that, in Persian lime, detoxification processes favored the maintenance of the phloemic flow in the plant and, thus, resulted in a better HLB-tolerance (Sivager et al., 2021). In Persian lime leaves, greater malondialdehyde (MDA) contents and stronger ascorbate peroxidase (APX) and catalase (CAT) activities were measured, compared to Mexican limes.

The voltammetry of immobilized microparticles (VIMP) is a solid state electrochemical technique (Scholz and Meyer, 1998; Scholz et al., 2014), which is capable of providing analytical information on a variety of sparingly soluble solids (Doménech-Carbó et al., 2013). VIMP method has been applied to test antioxidant properties of fruits and vegetables (Komorsky-Lovrić and Novak, 2011), to screen impurities in herbal remedies (Doménech-Carbó et al., 2013), and to test the reactivity of plant compounds with ROS (Doménech-Carbó et al., 2015). The use of VIMP was previously described for defining electrochemolomic profiles at the taxonomic level (Doménech-Carbó et al., 2015) and for monitoring plant defense responses to external stress (Doménech-Carbó et al., 2015). An evaluation of the voltammetric response in 2x and 4x Swingle citrumelo rootstock of infected and control trees in order to decipher the induced oxidative stress was performed. In **Figure 5A**, the voltammetric response can be interpreted in terms of the reduction (C_1_) and oxidation (A_1_) of electroactive components of 2x Swingle citrumelo grafted with Mexican lime that became totally or partially inactivated upon infection, so that the C_1_ wave is abruptly lowered. In 2x Swingle citrumelo grafted with Persian lime (**Figure 5B**), the dramatic decrease of the signal in the CLas+ rootstocks suggests that (i) antioxidant components oxidizable at high potentials are promoted in Persian lime/2x Swingle citrumelo relative to those in Mexican lime/2x Swingle citrumelo, and (ii) such antioxidant components are quite sensitive to the infection.

Cyclic voltammograms in roots of 4x Swingle citrumelo grafted with Mexican lime showed signals at potential clearly different than the signal C_1_ recorded with the 2x Swingle citrumelo CVs (**Figure 5C**). Accordingly, these signals can be attributed to the reduction of different natural products. The voltammograms of the infected rootstocks show a decrease in the intensity of the peak C_2_ and the disappearance of the peak C_3_, which is apparently substituted by a cathodic signal at −1.45 V(C_4_). These results are an agreement with a higher tolerance to HLB of 4x Swingle citrumelo, compared to 2x rootstocks. In this case, the A_2_ plus OER signal is high, but compared to the 2x Swingle citrumelo/Persian lime, the infection does not decrease its intensity (**Figure 5C**). As these are reduction peaks, the compound(s) responsible for the signals C1, C2, and C3 are not antioxidants (they should be easily oxidized); rather, these compounds would be “antireductants.”

In 4x Swingle citrumelo grafted with Persian lime, the cyclic voltammograms recorded at GCEs modified with the films of roots of 4x Swingle citrumelo (**Figure 4D**) showed that the cathodic region of the voltammograms of 4x Swingle citrumelo forms clearly differ from that of the 2x forms. This cathodic region was much more sensitive to infection by HLB than the corresponding region of the 2x ones were. Thus, this strong modification confirms the higher reactivity of 4x Swingle citrumelo against HLB infection. The anodic regions of the infected grafted forms (2x and 4x) experience sharp differences with the anodic region of the corresponding pristine forms.

Polyphenol contents analysis in 2x and 4x Swingle citrumelo rootstocks were very contrasted (**Figure 6**). If in CLas- root samples polyphenol contents were quite low, 4x presented higher contents, than those in 2x, whatever the scion. Those results would be in agreement with preadaptation to stress by limiting oxidative stress as previously proposed for polyploid genotypes (Allario et al., 2013; Khalid et al., 2021; Lourkisti et al., 2022). Interestingly, HLB infections led to a 10-fold increase of the polyphenol contents in 4x rootstocks, compared to 2x. As polyphenols play a crucial role in plant–environmental adaptation because of their role in biotic (Cheynier et al., 2013) and abiotic stress (Cheruiyot et al., 2007; Šamec et al., 2021; Tuladhar et al., 2021) defense, which is commonly attributed to their antioxidant activity (Martinelli et al., 2016), polyphenol may contribute to the better adaptation of polyploidy to HLB. The increase was even stronger when 4x rootstock was grafted with triploid Persian lime, underlining the importance of the polyploid rootstock-scion interactions as a tool to limit the oxidative stress induced by HLB. Finally, due to the TEM investigations performed in the root phloem cells, the presence of CLas in infected 2x and 4x Swingle citrumelo secondary roots was clearly demonstrated. However, this method was not adapted to quantify the bacterium within the tissues and did not indicate a link between cell wall degradation and membrane perturbation to detoxification processes, as was observed in the 4x root (**Figure 4**).

## Conclusion

**Figure 7:**
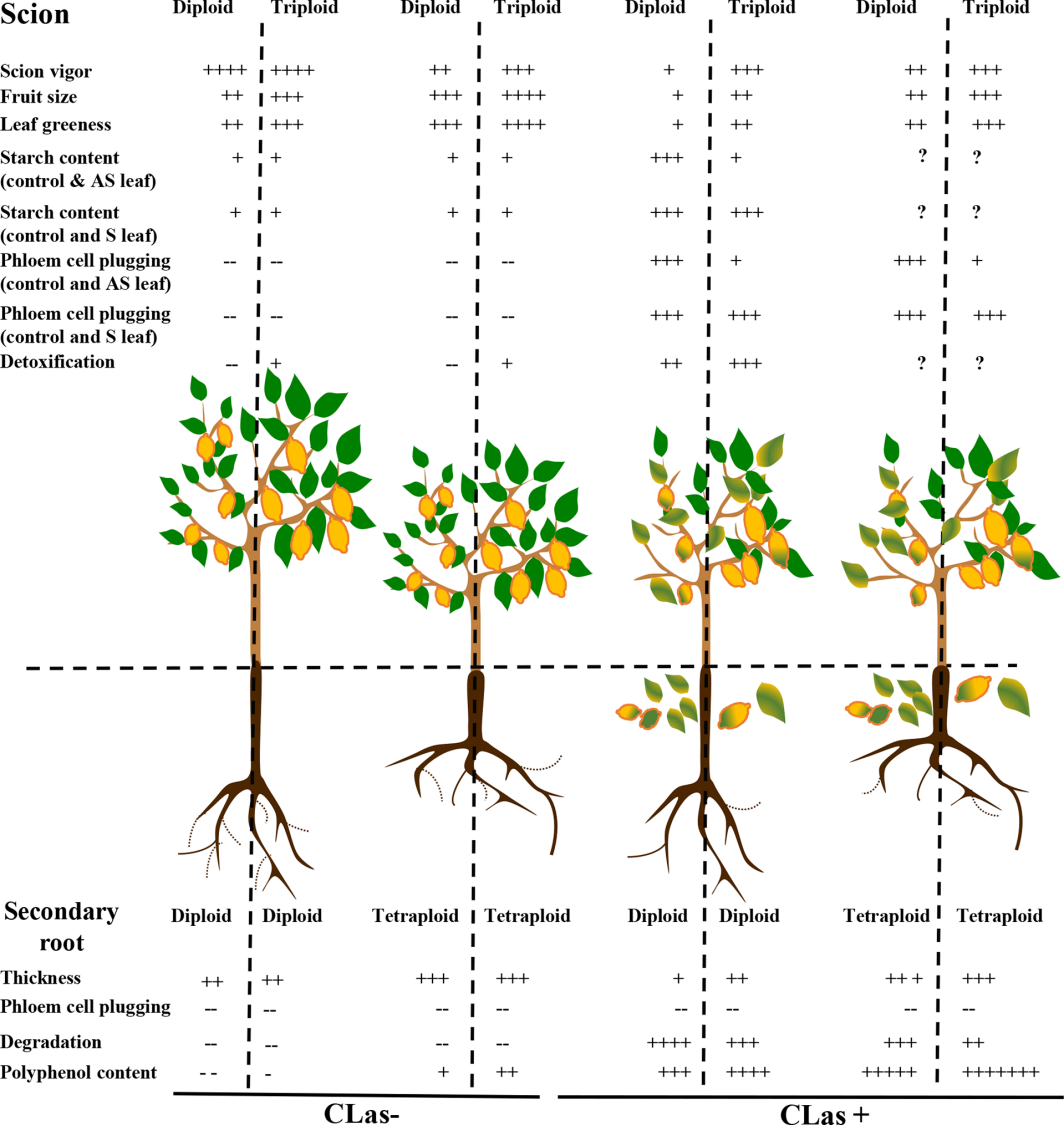
Impact of polyploidy at the rootstock and scion levels regarding different phenotypic traits modified by HLB. Scion/rootstock associations are evaluated in control (CLas-) and HLB infected trees (CLas+). AS leaf means asymptomatic leaf, and S leaf means symptomatic leaf. Data in the 2x and 3x scions grafted onto 2x rootstock are extracted from (Sivager et al., 2021).

In the field, the Persian grafted onto 2x Swingle citrumelo showed to be much more tolerant to HLB, compared to the Mexican lime (Sivager et al., 2021). In this present article, we investigated the same scion-varieties grafted onto 2x and 4x Swingle citrumelo, and we focused our investigation at the root compartment. Anatomical, physiological, and biochemical differentiations due to ploidy variation explain a large part of the better behavior of 3x scion/(4x/2x) Swingle citrumelo rootstock associations (**Figure 7**). Indeed, if the pores of 2x and 4x phloem root cells were not plugged by callose in CLas+ samples, oxidative stress in 2x roots was much more damaging and was associated with lower polyphenols contents, which may explain the greater root degradation observed in 2x scion/2x Swingle citrumelo rootstock associations. Interestingly, these findings demonstrate that the scion strongly impacts the tolerance to HLB, which is improved when associated with 4x Swingle citrumelo rootstock. To confirm the interest of polyploidy as a source of better tolerance to HLB, it would be required to investigate other 4x rootstock genotypes and 3x scion varieties. Investigations are also needed to decipher the molecular determinants of the better HLB-tolerance of 4x Swingle citrumelo rootstock and 3x Persian lime scion, as well as the polyploid scion/rootstock interactions.

## Data availability statement

The original contributions presented in the study are included in the article/**Supplementary Materials**. Further inquiries can be directed to the corresponding author.

## Author contributions

GS, LC, SB, RB, and BH performed the experiments and collected the physiological data. GS, AD-C, GC-T, and RM performed the statistical analyses, interpreted the results, and drafted the manuscript. GS and OG performed the analyses by SEM and TEM. BH and OG helped to draft the manuscript. All authors contributed to the article and approved the submitted version.

## Funding

GS and LC were supported by the “Collectivité Territoriale de Martinique” and the “Région Guadeloupe.” Investigations were supported by the “TROPICSAFE” project funded by European Union’s Horizon 2020 research and innovation program under grant agreement No. 727459 and the “CAVALBIO” project funded by FEDER and the Guadeloupe Region (2015–2021).

## Acknowledgments

The data presented have been presented in the D.3.4 report (Morillon et al., 2022) of the “TROPICSAFE” project that granted our research (European Union’s Horizon 2020 research and innovation program under grant agreement No. 727459).

## Conflict of interest

The authors declare that the research was conducted in the absence of any commercial or financial relationships that could be construed as a potential conflict of interest.

## Publisher’s note

All claims expressed in this article are solely those of the authors and do not necessarily represent those of their affiliated organizations, or those of the publisher, the editors and the reviewers. Any product that may be evaluated in this article, or claim that may be made by its manufacturer, is not guaranteed or endorsed by the publisher.
