# Supplementary figures and images for: Better tolerance to Huanglongbing is conferred by tetraploid Swingle citrumelo rootstock and is influenced by the ploidy of the scion
Source: Front Plant Sci. 2022 Nov 3;13:1030862. doi: 10.3389/fpls.2022.1030862 (PMC9669798; doi:10.3389/fpls.2022.1030862)

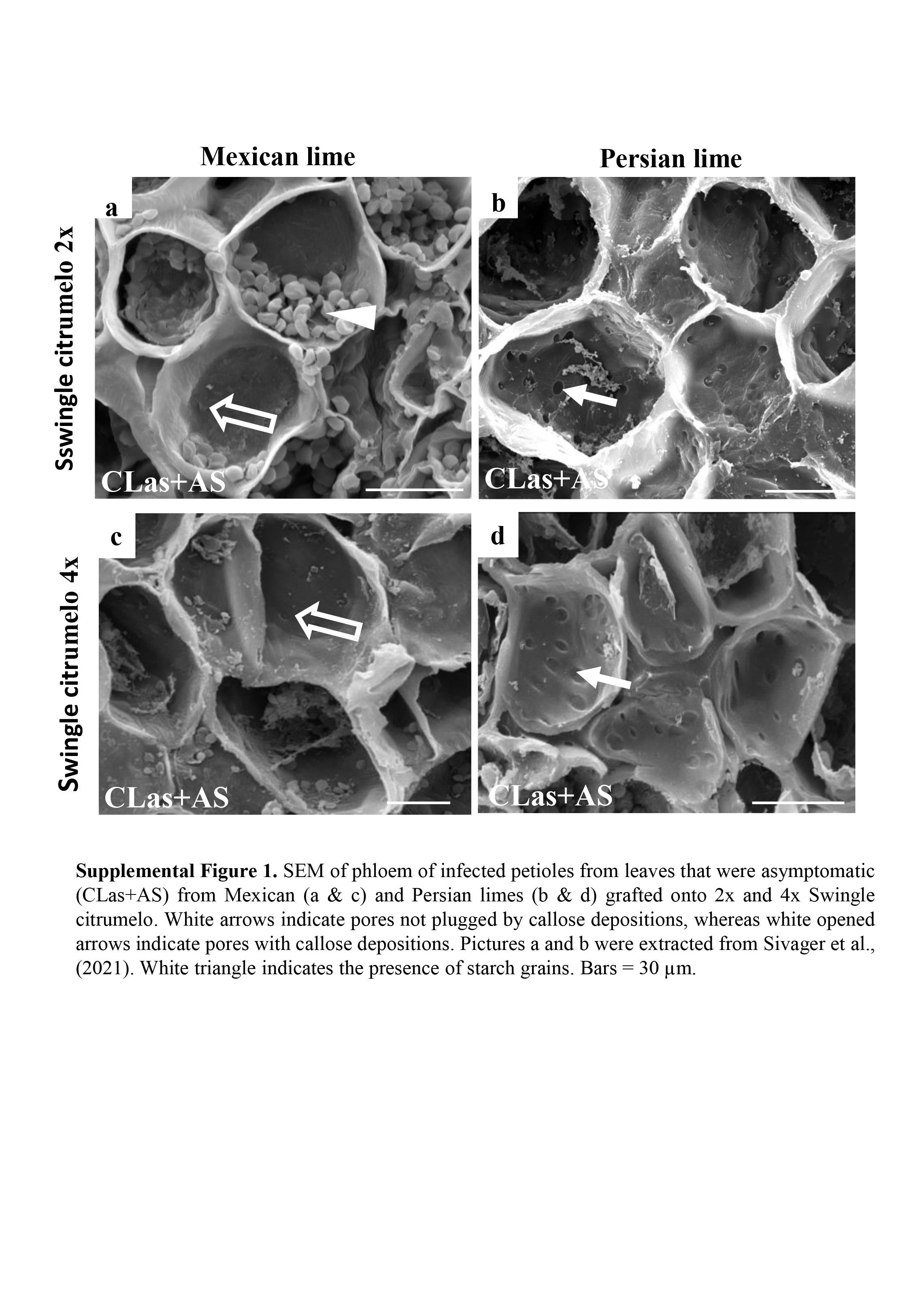

Supplement: Supplementary file 1 [file Image_1.tif]

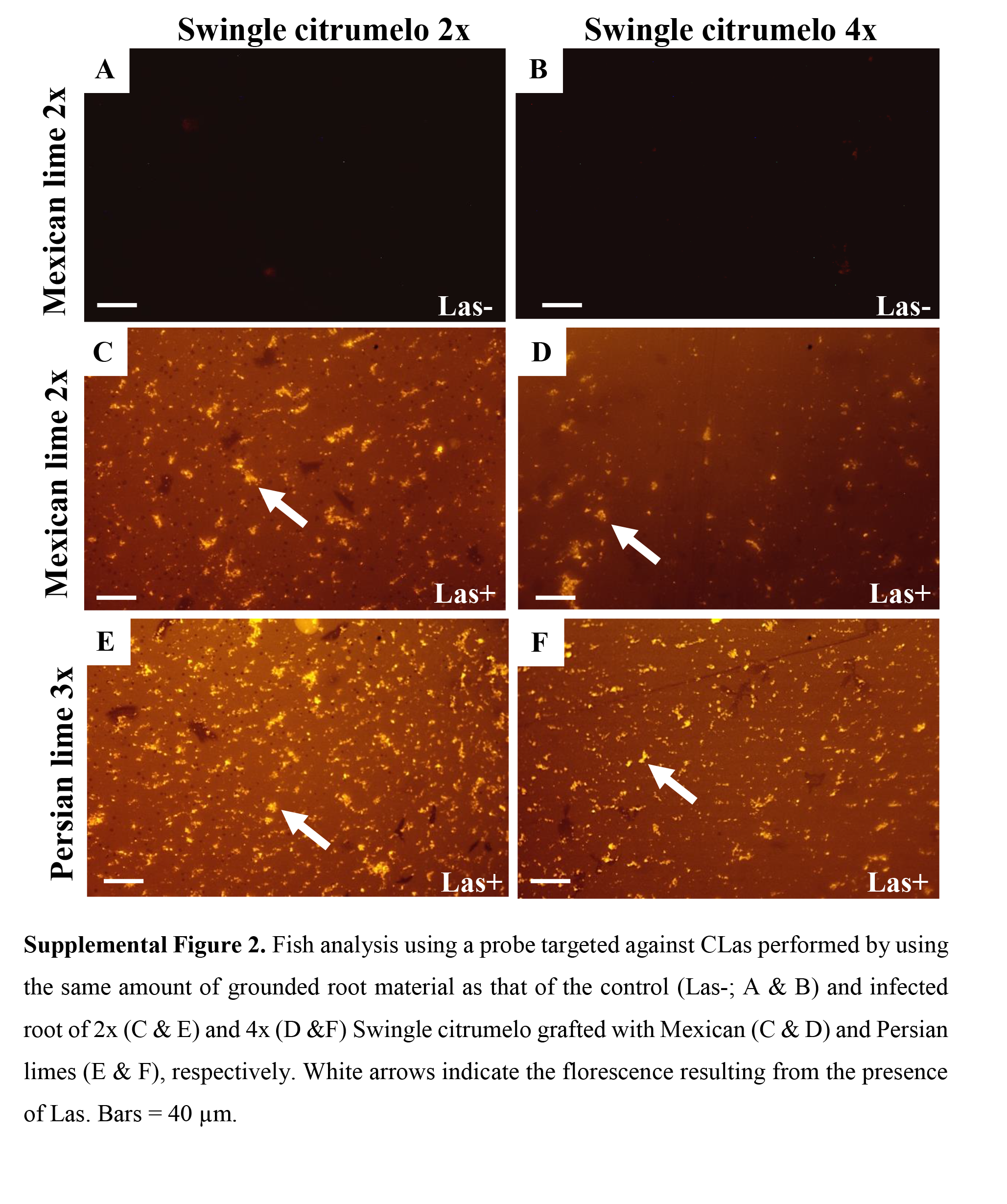

Supplement: Supplementary file 2 [file Image_2.tif]

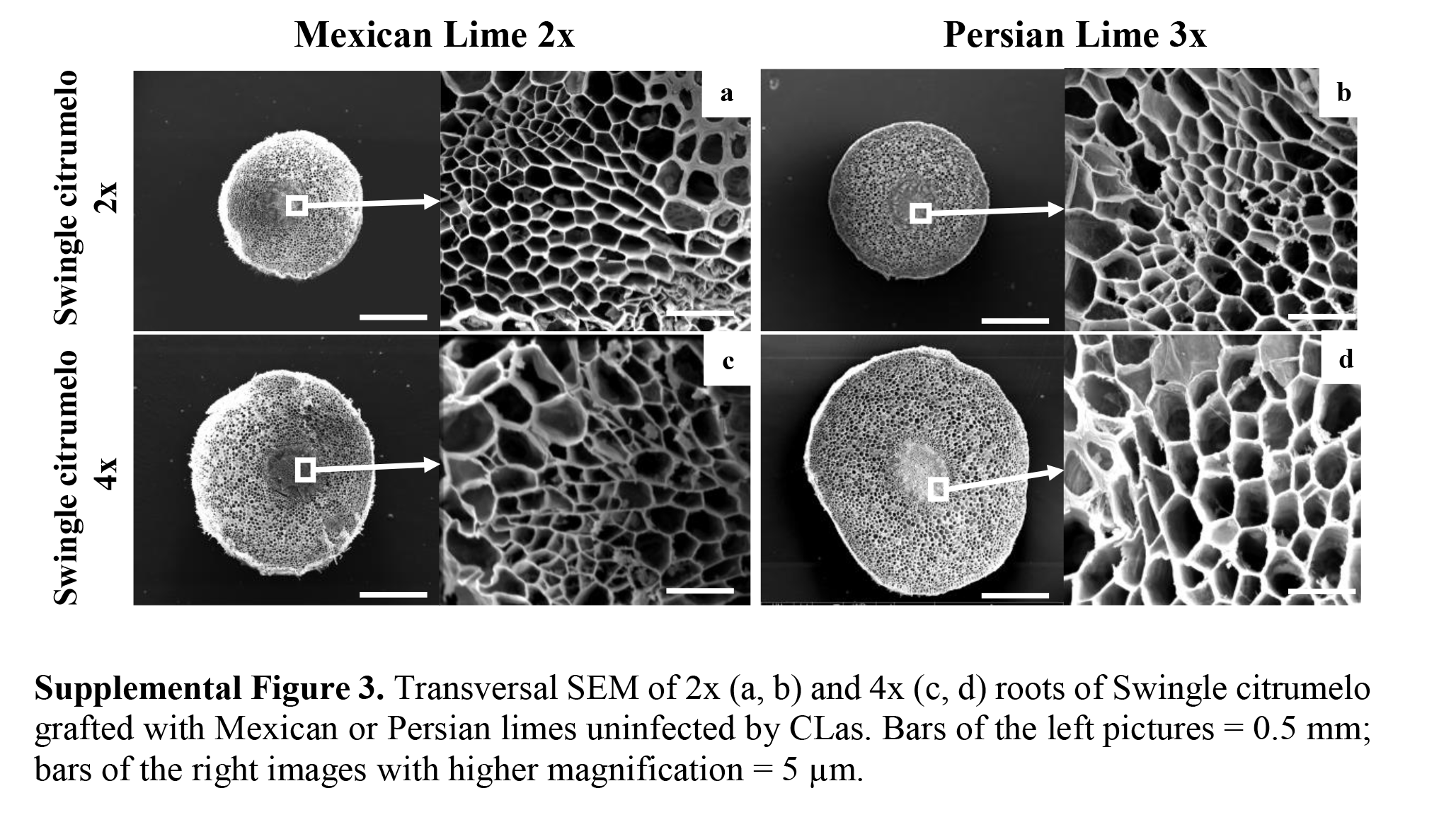

Supplement: Supplementary file 3 [file Image_3.tif]

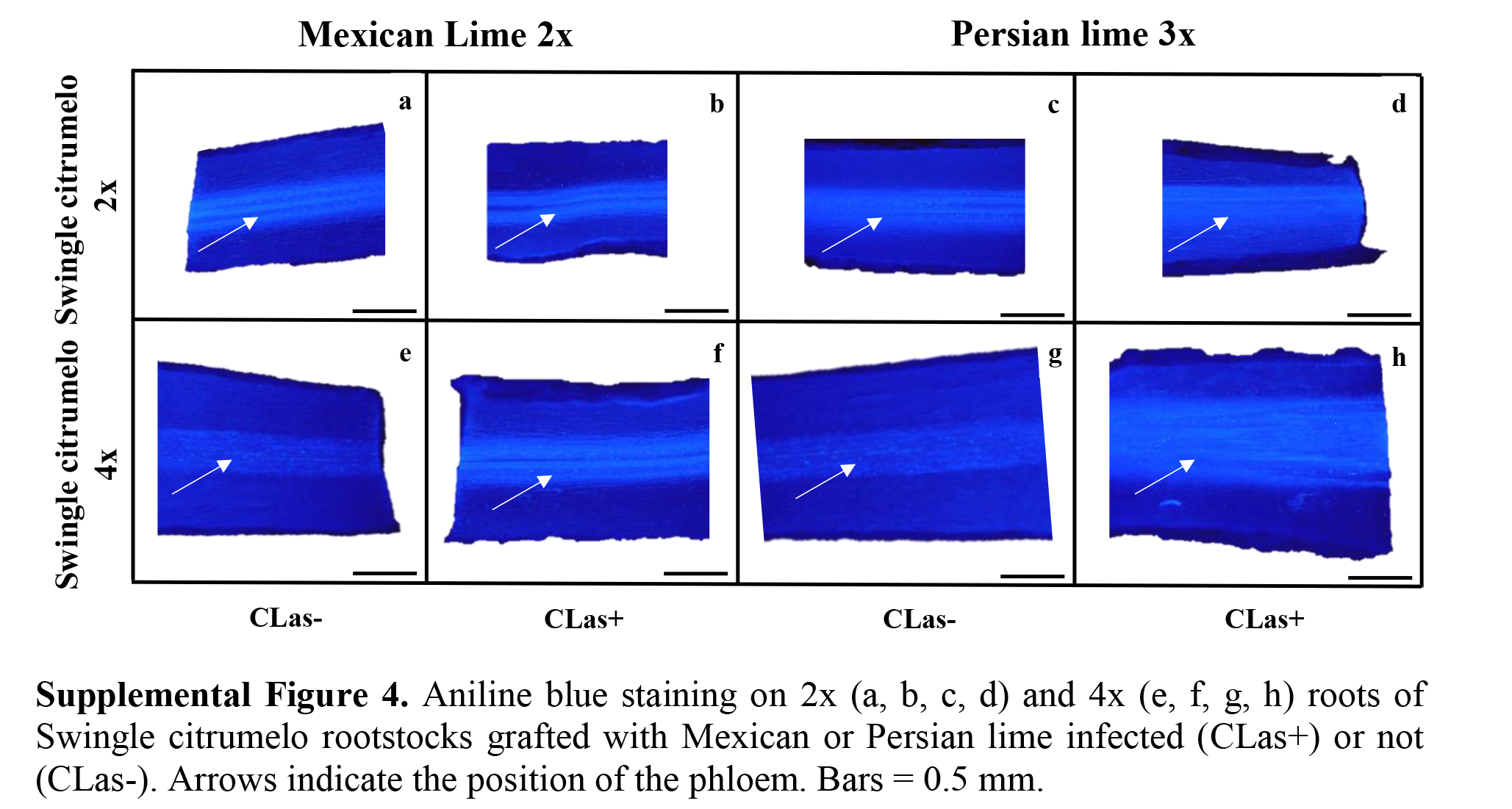

Supplement: Supplementary file 4 [file Image_4.tif]
